# Cardiovascular Risk Assessment: A Comparison of the Framingham, PROCAM, and DAD Equations in HIV-Infected Persons

**DOI:** 10.1155/2013/969281

**Published:** 2013-10-21

**Authors:** Max Weyler Nery, Celina Maria Turchi Martelli, Erika Aparecida Silveira, Clarissa Alencar de Sousa, Marianne de Oliveira Falco, Aline de Cássia Oliveira de Castro, Jorge Tannus Esper, Luis Carlos Silva e Souza, Marília Dalva Turchi

**Affiliations:** ^1^Postgraduate Studies Program, Institute of Tropical Pathology and Public Health, Federal University of Goiás, Brazil; ^2^Department of Medicine, Catholic University of Goiás, Brazil; ^3^Institute of Tropical Pathology and Public Health, Federal University of Goiás, Brazil; ^4^National Institute for Health Technology Assessement, Brazil; ^5^School of Nutrition, Federal University of Goiás, Brazil; ^6^Postgraduate Studies Program in Health Sciences, School of Medicine, Federal University of Goiás, Brazil

## Abstract

This study aims to estimate the risk of cardiovascular disease (CVD) and to assess the agreement between the Framingham, Framingham with aggravating factors, PROCAM, and DAD equations in HIV-infected patients. A cross-sectional study was conducted in an outpatient centre in Brazil. 294 patients older than 19 years were enrolled. Estimates of 10-year cardiovascular risk were calculated. The agreement between the CVD risk equations was assessed using Cohen's kappa coefficient. The participants' mean age was 36.8 years (SD = 10.3), 76.9% were men, and 66.3% were on antiretroviral therapy. 47.8% of the participants had abdominal obesity, 23.1% were current smokers, 20.0% had hypertension, and 2.0% had diabetes. At least one lipid abnormality was detected in 72.8%, and a low HDL-C level was the most common. The majority were classified as having low risk for CV events. The percentage of patients at high risk ranged from 0.4 to 5.7. The PROCAM score placed the lowest proportion of the patients into a high-risk group, and the Framingham equation with aggravating factors placed the highest proportion of patients into the high-risk group. Data concerning the comparability of different tools are informative for estimating the risk of CVD, but accuracy of the outcome predictions should also be considered.

## 1. Introduction

Highly active antiretroviral therapy (HAART) has significantly reduced morbidity and mortality among AIDS patients in many parts of the world [1, 2]. In addition, broad coverage of HAART is also associated with the reduction in the risk of HIV transmission at the population level [3]. The World Health Organization and the Joint United Nations Program on HIV/AIDS have set an international goal for expanding HAART to 15 million people by 2015 [4]. Brazil has a well-established HIV control program, and HAART has been universally offered to all eligible individuals at no charge since 1996. By 2011, approximately 200,000 HIV-infected patients were receiving antiretroviral therapy (ART), resulting in a remarkable survival benefit in the last decade [5, 6].

HAART has enhanced the life expectancy and improved the quality of life for HIV-infected patients. However, the aging of the population and the cumulative adverse effects caused by long periods of exposure to antiretroviral drugs have markedly increased the prevalence of dyslipidaemia, insulin resistance, impaired glucose metabolism, and abnormal fat distribution among HIV patients [7, 8]. Patients with metabolic syndrome have an increased risk of developing chronic nontransmissible conditions such as diabetes and cardiovascular diseases [9, 10].

Overall, the benefits of HAART in reducing mortality significantly outweigh the risks of metabolic abnormalities. Nevertheless, reducing metabolic abnormalities and identifying groups at high risk for cardiovascular diseases (CVD) are an important part of HIV management. Guidelines about managing dyslipidaemia in the general population [11] and in HIV-infected patients [12, 13] recommend identifying and treating patients at high risk for cardiovascular events. Traditional cardiovascular risk factors, such as smoking, lack of physical activity, and metabolic syndrome, play a very important role in determining cardiovascular events. Assessing and addressing those modifiable risk factors are important for CVD prevention.

Over the past decade, an increasing number of studies have assessed the cardiovascular risk profile of HIV-infected patients [14–22]. The majority of the studies used cardiovascular risk equations developed for a non-HIV-infected population, such as the Framingham or the Prospective Cardiovascular Münster Study (PROCAM) scores. The accuracy of these risk scores for predicting cardiovascular events in HIV-infected patients is not well established. Generally, these scores have been proposed and validated in high-income populations of older patients. In the last decade, a growing number of studies estimated CVD risk in HIV-infected populations, mainly using the Framingham score and less frequently using other cardiovascular risk equations, such as the PROCAM and Systematic Coronary Risk Evaluation (SCORE) equations [14–17, 19–25]. The prevalence of patients at high risk for coronary events in the next 10 years varies largely among these studies, ranging from less than 1% to 21% [15, 16]. These equations were not developed to assess cardiovascular risk in HIV-infected patients, and their accuracy is still uncertain [26, 27].

Presently, there is only one cardiovascular risk score specifically developed for HIV-infected patients [18]. It was based on the results of a large multicentre cohort study (The Data Collection on Adverse Effects on Anti-HIV Drugs Cohort-DAD), conducted mainly in Europe and North America. The DAD equation takes antiretroviral drug exposure into account as a potential risk factor for cardiovascular events. To date, the DAD risk equation has only been applied for CVD risk estimation in one developing country [22]. This study aims to estimate the cardiovascular risk for HIV-infected patients in Brazil using the Framingham, PROCAM, and DAD equations and to compare the results.

## 2. Methods

This study is a cross-sectional analysis of a cohort of HIV-infected patients (PRECOR study) attending an outpatient public referral centre for infectious diseases in central Brazil. HIV-infected patients between 20 and 75 years old were consecutively enrolled between October 2009 and January 2011. The study protocol was approved by the local Institutional Review Board. The participants showed no clinical evidence of active opportunistic diseases at the time of enrolment.

*Clinical and Epidemiological Investigation.* At the time of enrolment, all the participants were interviewed and examined by the same cardiologist, a member of the research team. A structured questionnaire that addressed sociodemographic variables and general medical history was used to collect data.

 The participants were also asked about the time of their HIV diagnosis, previous opportunistic diseases, antiretroviral exposure, and cardiovascular risk factors. Smoking status was classified as never, former, or current (those who had stopped smoking within the past 30 days were considered current smokers). The participants were considered to have a history of cardiovascular disease if they had a convincing history of myocardial infarction (MI), angina, stroke, peripheral arterial disease, or an intervention for coronary artery disease. Family history was defined as cardiovascular disease in a first-degree male before the age of 55 or in a first-degree female relative before the age of 65. 

Arterial blood pressure was measured. The patients on antihypertensive therapy or with systolic blood pressure ≥140 mmHg and/or diastolic blood pressure ≥90 mmHg were classified as having hypertension [28].

Afterward, the participants met with a multidisciplinary research team including nutritionists, a pharmacist, a biomedical professional, anthropometrists, and a physiotherapist. Waist circumference (WC) was measured as the narrowest circumference between the lower rib margin and the anterior superior iliac crest using a standard inelastic anthropometric tape. A WC ≥94 centimetres (cm) for men and ≥80 cm for women was considered a risk factor for CVD [29].

*Laboratory Investigation*. The participants were advised to fast overnight for 12 h, to avoid alcohol consumption for 3 days prior to blood collection. All of the analyses were performed at the same laboratory.

Serum total cholesterol (TC), high-density lipoprotein (HDL) cholesterol and triglycerides (TG) were assayed. Serum low-density lipoprotein (LDL) cholesterol was estimated using the Friedewald formula when TG was ≤400 mg/dL [30]. Dyslipidaemia was defined as LDL cholesterol ≥160 mg/dL, HDL cholesterol <40 mg/dL for men and <50 mg/dL for women or diabetics or triglycerides ≥150 mg/dL [11, 31].

High sensitivity C-reactive protein (hsCRP) was quantified. hsCRP values <1 mg/L were classified as normal, between 1 and 3 mg/L were classified as intermediate risk, and >3 mg/L were classified as high risk [32].

Diabetes was defined as a fasting serum glucose level greater than 125 mg/dL [33] or a self-reported diagnosis of diabetes and use of specific therapy. The glomerular filtration rate (GFR) was calculated based on the Cockcroft-Gault equation [34]. A GFR below <60 mL/min or a serum creatinine level equal to or higher than 1.5 mg/dL indicated impaired renal function [35].

 A urine sample was collected to determine the albumin/creatinine ratio (ACR) [36]. Patients were categorised as having normoalbuminuria (<30 mcg/mg), microalbuminuria (30 to 299 mcg/mg), or clinical albuminuria (>300 mcg/mg) [37].

CD4 counts and the HIV RNA levels were obtained from the patient's medical chart. HAART data were obtained from the patients' medical and pharmaceutical records. 

Metabolic syndrome was defined in accordance with the standards of the International Diabetes Federation [29].

*Calculation of Cardiovascular Risk*. Framingham and PROCAM scores were calculated to predict 10-year CVD risk. DAD equations were used to calculate 5-year CVD risk. The participants with a history of CVD or were older than 75 were excluded from this analysis.

*Framingham Equation*. The following variables were included in the equation: age, sex, systolic blood pressure, antihypertensive therapy (yes or no), serum TC and HDL-cholesterol values, and current smoking status (yes or no). The 10-year risk of CVD was classified as low (<10%), moderate (10% to 20%), or high (>20%) [38]. The patients with diabetes were classified as high risk [11, 31]. The participants who had low or moderate 10-year risk according to the Framingham equation were also reevaluated based on the presence of aggravating factors: family history of CVD, metabolic syndrome, serum creatinine ≥1.5 mg/dL, hsCRP >3.0 mg/L, or albuminuria >30 mcg/mg. Patients presenting at least one of these aggravating factors were reclassified into the high-risk category for CVD over 10 years (Framingham equation with aggravating factors, according to the IV Brazilian Guideline for Dyslipidemia and Atherosclerosis Prevention) [31].

*PROCAM Equation*. The following variables were included in the equation: age; sex; known diabetes or fasting blood glucose level ≥120 mg/dL; systolic blood pressure; serum TG, LDL, and HDL-cholesterol levels; current nicotine consumption and family history of CVD. The 10-year risk of acute coronary events was classified as low (<10%), moderate (10% to 20%), or high (>20%) [39].

*DAD Equation*. The following variables were included in the equation: age; sex; systolic blood pressure; serum CT and HDL-cholesterol level; diabetes; smoking status; family history of CVD; current use of abacavir, indinavir, or lopinavir; and the number of years on indinavir or lopinavir. The 5-year risk of coronary heart disease was classified as low (<1%), moderate (1 to 5%), high (5 to 10%), or very high (>10%) [18].

*Statistical Analysis*. Normally distributed continuous variables were expressed as means and standard deviations; otherwise, they were expressed as medians and interquartile ranges (IQR). Categorical variables were expressed as percentages with 95% confidence intervals (95% CI). A *χ*² test or Fisher's test was used to compare proportions.

The agreement between the CVD risk equations was assessed using Cohen's kappa coefficient with 95% CIs. The participants at high or very high risk according to the DAD equation were considered high-risk patients for the comparison with the Framingham, Framingham with aggravating factors, and PROCAM equations.

Two-sided *P* values <0.05 were considered statistically significant. The data were analysed using IBM SPSS Statistics base 18.0 for MAC (Chicago, IL, USA).

## 3. Results

A total of 335 HIV-infected adults were enrolled during the study period. Clinical and laboratory data were obtained from 299 patients (89.3%). Thirty-six participants (10.7%) were excluded because their laboratory analyses were incomplete. There was a higher proportion of previous intravenous drug users (IDU) among the individuals with incomplete laboratory data (*P* < 0.01). There were no statistically significant differences in sex, age, educational level, or monthly income (*P* > 0.05) between these two groups. Five patients reported a previous cardiovascular event and were excluded, resulting in 294 participants. 

Table 1 presents the sociodemographic and clinical characteristics of the 294 participants. The study population was predominantly male (76.9%). The mean age was 36.8 (SD = 10.3), and the ages ranged from 20 to 71 years. Approximately 80% (185/226) of the males were younger than 45, and approximately 90% (59/68) of the females were younger than 55. The majority of the participants (72.4%) had more than 8 years of schooling. A total of 92.2% reported being exposed to HIV through a sexual route; among these participants, 54.1% declared that they were homosexual males or bisexual. Previous IDU was reported by 2.7%.

The median time since receiving the HIV diagnosis was 2.0 years (IQR: 0.7–5.0 years). At least one opportunistic disease in the previous 12 months was reported by 5.8% of those recruited. At the time of enrolment, 54.1% presented an undetectable HIV viral load, and 72.6% had a CD4 count ≥350 cells/mm^3^. Overall, 195 (66.3%) out of 294 participants were receiving HAART, with a median duration of 19.0 months (IQR: 5.0–54.0 months) (Table 1).

**Figure 1:**
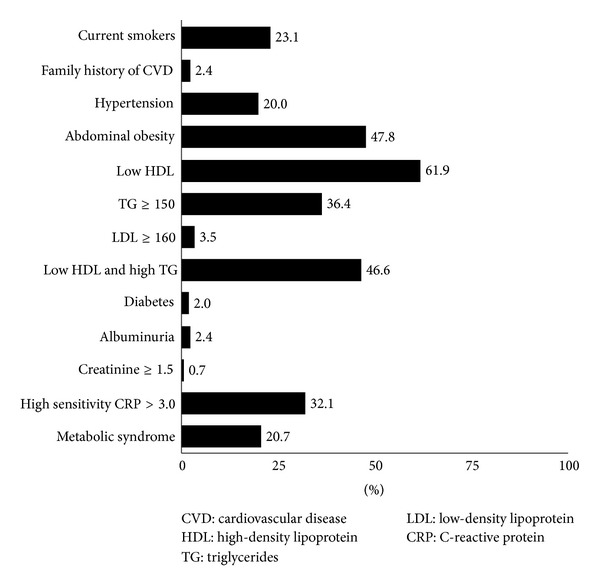
Prevalence of cardiovascular risk factors among 294 HIV-infected patients.

Figure 1 shows the prevalence of cardiovascular risk factors among the participants. A history of smoking was reported by 46.9% (138/294), and 23.1% (68/294) were current smokers. Seven participants reported a family history of premature atherosclerotic disease. Twenty-three patients had high blood pressure (HBP), and 47.8% of them were receiving antihypertensive therapy. At the time of the physical examination, 55 participants had HBP. In total, 20.0% (59/294) of the participants were considered hypertensive. Abdominal obesity was detected in 47.8% of the participants. The proportion of females with abdominal obesity (82.4%) was higher than the proportion of males (23.0%) (*P* < 0.001).

Most (72.8%) participants had at least one abnormality in their lipid profile. The most common dyslipidaemia was a low HDL-C level, which was found in 61.9% of the participants. The prevalence of isolated hypertriglyceridemia and isolated hypercholesterolemia was 36.4% and 3.5%, respectively. The prevalence of low HDL-C combined with high TG was 46.6%. For 11 participants, LDL cholesterol was not estimated because TG was higher than 400 mg/dL. The mean values were 169.5 (SD = 42.7) mg/dL for TC, 42.5 (SD = 11.3) mg/dL for HDL cholesterol, 96.6 (SD = 32.5) mg/dL for LDL cholesterol, and 151.4 (SD = 104.5) mg/dL for TG.

A total of 6 patients (2.0%) were categorised as having diabetes. Seventeen patients had albuminuria, and two had creatinine levels equal to or higher than 1.5 mg/dL. High sensitivity C-reactive protein >3 mg/L was detected in 32.1% of the participants. Sixty-one (20.7%) participants had metabolic syndrome. The prevalence of metabolic syndrome was higher among females (36.8%) than among males (16.0%) (*P* < 0.001). 

Table 2 presents the antiretroviral regimens at the baseline interview by 195 patients receiving treatment. Of the participants, 62.6% were taking a combination of zidovudine, lamivudine, and efavirenz. The second most frequent antiretroviral regimen was a combination of zidovudine, lamivudine, and lopinavir boosted by ritonavir (12.8%). Twenty-nine patients (15.1%) were using a regimen containing tenofovir. Fifty-one (26.2%) patients were using a protease inhibitor (PI) regimen; among these participants, 28 were taking a lopinavir regimen (25 were taking a regimen boosted by ritonavir). Additional 6 patients used lopinavir in a previous regimen. The median duration of lopinavir-based therapy was 13.0 months (IQR: 4.0–23.5). One participant was taking abacavir. None of the participants were ever exposed to indinavir.

**Figure 2:**
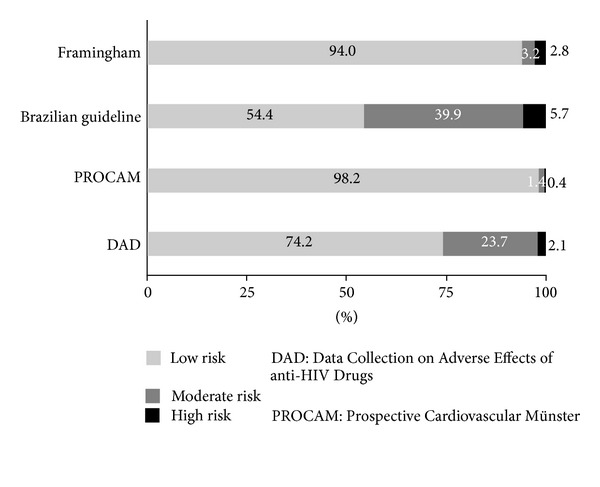
Prevalence of estimated CVD risk according to Framingham, Framingham with aggravating factors (Brazilian Guideline for Dyslipidemia and Atherosclerosis Prevention), PROCAM and DAD risk equations for 283 HIV-infected patients.

Figure 2 presents the estimated CVD risk of the 283 HIV-infected patients based on the Framingham, Framingham with aggravating factors, PROCAM, and DAD risk equations. Eleven participants were excluded because they had TG >400 mg/dL. The majority of participants were classified at low risk for future CV events. The percentage of patients classified as having a high risk of future CVD ranged from 0.4 to 5.7 using these four scoring systems.

*Framingham Equation.* Based on the Framingham equation, 94.0% of the participants were classified as low-risk, 3.2% as moderate-risk, and 2.8% as high-risk patients.

*Framingham with Aggravating Factors.* Of the participants, 54.4% were classified as low-risk patients, 39.9% as moderate-risk patients, and 5.7% as high-risk patients for CVD. At least one aggravating cardiovascular risk factor was detected in 42.0% of the participants. The most frequent aggravating factor was hsCRP >3.0 mg/L (32.1%), followed by the presence of metabolic syndrome (20.7%). Impaired renal function was identified in 0.7% of the participants, and 2.4% reported a family history of CVD.

*PROCAM Equation*. Of the participant, 98.2% were classified as low-risk patients, 1.4% as moderate-risk patients, and 0.4% as high-risk patients for CVD.

*DAD Equation*. Of the participants, 74.2% were classified as low-risk patients, 23.7% as moderate-risk patients, and 2.1% as high-risk patients. None had a very high risk for CVD. 

The agreement of the DAD equation with the Framingham equation was 77.4% (*k* = 0.23; 95% CI 0.07–0.39), with the Framingham with aggravating factors was 56.7% (*k* = 0.14; 95% CI 0.02–0.25), and with the PROCAM equation was 75.3% (*k* = 0.07; 95% CI 0.00–0.26). The agreement was good, but low kappa values were detected for all three comparisons (Table 3).

## 4. Discussion

The present study was conducted in a population of HIV-infected individuals, mainly composed of young adult males, who were clinically stable. One-third of them were naïve to antiretroviral drugs. Less than 3.0% of the patients were at high risk for cardiovascular events over the next 10 years according to equations developed for the general population, such as the Framingham and PROCAM equations. A similar profile was obtained using the DAD equation, which was specifically constructed for HIV patients. When aggravating factors were incorporated into the Framingham equation, as recommended by the Brazilian Guidelines for clinical management, the proportion of the participants classified in the high-risk group increased twofold: almost 40% of the patients were classified in the intermediate-risk group for CV events. Similarly, the DAD equation placed a higher proportion of the patients in the moderate-risk category. 

Initially, CVD risk equations were conceived for those over 30 in the general North American and European populations. However, these risk estimations were applied to younger age groups in other settings and, more recently, to people living with HIV [15]. In alignment with our results, other Latin American studies also found a low risk of future CV events using the conventional cardiovascular equations among predominantly male HIV patients in their 30s and 40s [16, 19]. In contrast, a high risk of cardiovascular events was found among older populations, those with greater exposure to antiretroviral drugs, or populations with a higher prevalence of well-known risk factors, such as smoking, diabetes, or hypertension [14, 15, 24].

In our study, the participants were young and had relatively short exposure to antiretroviral drugs. The prevalence of diabetes was low, but the prevalence of abdominal obesity was high, especially among the female participants. Furthermore, most of our patients had low incomes. Obesity and low income were associated with an increased 10-year risk for CVD in a large study conducted in the USA [23]. In addition, aging, abdominal obesity, and the duration of antiretroviral drug exposure are intrinsically associated with the development of diabetes [40], leading to an increased risk for CVD disease in the study population in the near future.

A high triglyceride level is one of the most common lipid abnormalities described among HIV-infected patients [40, 41]. A similar lipid profile was found in the current study and in a previous evaluation in the same setting [42]. One potential disadvantage of using Framingham scores for HIV-infected patients is that triglyceride values are not incorporated into the CVD risk equations. However, the use of elevated triglycerides as a marker for future CVD is still a controversial issue [43].

In the PROCAM equation, triglycerides and LDL levels are incorporated into the risk equation. Nevertheless, for patients with triglyceride levels higher than 400 mg/dL, it is not possible to estimate LDL levels, which hampers CVD risk calculation. Similar to our results, other studies using PROCAM equations [14, 15, 19, 24, 25] have found a lower percentage of patients with high 10-year risk for CV events compared with the risk estimates using the Framingham equation. In two studies [19, 25], the coefficient of agreement between the PROCAM and Framingham equations was evaluated. In one study [19], the low- and moderate-risk groups were combined to compare the Framingham and PROCAM scores, yielding weak agreement between these equations. Another study found good agreement between these scores [25]. Determining the proportion of patients with high or moderate 10-year cardiovascular risk has clinical and economic repercussions. It has not been determined which of these two equations is more accurate, and the answer most likely will not be determined [27]. Large cohort studies conducted in HIV-infected populations with different clinical and demographic profiles would be needed to approach this question [27].

In the present study, the Framingham equation with aggravating risk factors most likely overestimated the 10-year cardiovascular risk. The percentage of participants in the high-risk group increased from 2.8% to 5.7%, and the percentage of moderate-risk patients increased more than 10 times (from 3.2% to 39.9%), mainly because of the high sensitivity C-reactive protein (hsCRP) values. hsCRP is a nonspecific marker for inflammation and is considered an independent cardiovascular risk predictor [32]. HIV infection itself and opportunistic infections can lead to a proinflammatory state. The accuracy of CRP as a cardiovascular risk predictor has not been well established in HIV-infected populations [44].

Few large cohort studies have investigated the accuracy of the conventional risk equations to predict cardiovascular events in HIV-infected populations [18, 45, 46]. To date, the DAD equation is the only tool for estimating cardiovascular risk developed specifically for an HIV-infected population. In our study, the proportion of patients in the high-risk group for CV events was low using the DAD equation and conventional risk equations (Framingham and PROCAM). Another study from Thailand found a low prevalence of patients with high CVD risk using the DAD equation and the Framingham equation, with good agreement between these scores [22]. However, the Framingham equation predicted higher CVD risk compared with the DAD equation. In our results, the DAD equation placed a higher proportion of the patients in the moderate category of risk for CVD compared with the Framingham and PROCAM scores; this result has implications for clinical followup and management.

Methodological issues regarding the comparability and applicability of these different CVD risk scores, particularly among people living with HIV, have been thoroughly discussed, most recently by D'Agostino [27]. In addition to the differences in the composition of the cardiovascular risk scores and the differences in the relevant outcomes, the comparison of CVD scores using kappa coefficients may have some limitations. Our findings pointed out good agreement for the risk predictions produced by the DAD equations compared with Framingham or PROCAM in constrast with low statistical kappa estimates. Other studies also found good agreement between different risk systems but calculated low kappa values [22, 25]. This apparent paradox could be explained by the imbalanced distribution of the values and by kappa's dependence on the prevalence of the measured event [47]. However, the clinical significance of these inconsistencies is not completely understood.

## 5. Conclusions

Comparing different tools that predict the risk of CVD is informative, but these comparisons do not assess the accuracy of the outcome. Because HAART is offered to large cohort of HIV patients in Brazil, it seems promising to address this issue by monitoring patients and CV events using the existing official surveillance system. These data would be valuable for individual clinical management and for economic evaluations from a public health perspective. Large cohort studies including different HIV-infected groups would be necessary to address these questions.

## Figures and Tables

**Table 1:** Sociodemographic and clinical characteristics of 294 HIV-infected patients.

Characteristics	Values	95% CI
Sociodemographic		
Males	226 (76.9)	71.4–81.9
Age, years mean (SD)	36.8 (10.3)	—
Male <45 y	185 (81.9)	76.5–86.5
Female <55 y	59 (86.8)	78.7–94.8
≥8 y schooling	213 (72.4)	63.0–82.9
Exposure categories		
Sexual route	271 (92.2)	81.5–103.8
Homosexual or bisexual^a^	142 (51.4)	43.3–60.6
Previous IDU^b^	7 (2.7)	1.0–5.5
Clinical HIV history		
HIV diagnosis, years median (IQR)	2.0 (0.7–5.0)	—
Opportunistic disease in previous 12 months^c^	16 (5.8)	3.5–8.9
Undetectable HIV viral load^d^	153 (54.1)	48.1–60.2
CD4 ≥350 cells/mm^3e^	207 (72.6)	22.4–32.7
Antiretroviral therapy	195 (66.3)	61.3–71.7
ART duration, months, median (IQR)	19.0 (5.0–54.0)	—

IDU: intravenous drug users; ART: antiretroviral therapy.

^a^18 missing, ^b^33 missing, ^c^17missing, ^d^11 missing, ^e^9 missing.

Continuous variables are presented as means (SD), median (IQR), and categorical variables are presented as absolute value and percentage with 95% CI.

**Table 2:** Antiretroviral regimen in 195 HIV-infected patients.

Antiretroviral regimen	Class of drug	*N* (%)	95% CI
ZDV + 3TC + EFZ	2NRTI + 1NNRTI	122 (62.6)	51.9–74.7
ZDV + 3TC + LPV/RTV	2NRTI + 1PI/Booster	25 (12.8)	8.3–18.9
3TC + TDF + EFZ	2NRTI + 1NNRTI	15 (7.7)	4.3–12.7
3TC + TDF + ATV + RTV	2NRTI + 1PI/Booster	5 (2.6)	0.8–5.9
ZDV + 3TC + TDF + LPV + RTV	3NRTI + 1PI/Booster	3 (1.5)	0.3–4.5
ZDV + 3TC + NVP	2NRTI + 1NNRTI	3 (1.5)	0.3–4.5
Others	—	22 (11.3)	7.0–17.0

ATV: atazanavir; ZDV: zidovudine; 3TC: lamivudine; EFZ: efavirenz; LPV: lopinavir; NNRT: nonnucleoside reverse transcriptase inhibitor; NRTI: nonnucleoside reverse transcriptase inhibitor; NVP: nevirapine; PI: protease inhibitor; RTV: ritonavir; TDF: tenofovir.

**Table 3:** Comparison between CVD risk estimation using different equations (283 patients).

	Cardiovascular risk	Framingham			Brazilian Guideline			PROCAM		
		Low	Moderate	High	Low	Moderate	High	Low	Moderate	High
DAD	Low	210	0	0	126	85	0	210	0	0
	Moderate	56	6	5	28	29	10	65	2	0
	High and very high	0	3	3	0	0	6	3	2	1
Agreement			77.4%			56.7%			75.3%	
Kappa			0.23			0.14			0.07	
95% CI			0.07–0.39			0.02–0.25			0–0.26	

DAD: Data Collection on Adverse Effects of Anti-HIV Drugs; PROCAM: Prospective Cardiovascular Münster; CI: confidence Interval.
